# Population exposure to hazardous air quality due to the 2015 fires in Equatorial Asia

**DOI:** 10.1038/srep37074

**Published:** 2016-11-16

**Authors:** P. Crippa, S. Castruccio, S. Archer-Nicholls, G. B. Lebron, M. Kuwata, A. Thota, S. Sumin, E. Butt, C. Wiedinmyer, D. V. Spracklen

**Affiliations:** 1 COMET, School of Civil Engineering and Geosciences, Newcastle University, Newcastle upon Tyne, NE1 7RU, UK; 2 School of Mathematics and Statistics, Newcastle University, Newcastle upon Tyne, NE1 7RU, UK; 3 Atmospheric Chemistry Observations & Modeling Laboratory, National Center for Atmospheric Research, Boulder, CO, 80301, USA; 4 Earth Observatory of Singapore, Nanyang Technological University, 639798, Singapore; 5 Asian School of the Environment, Nanyang Technological University, 639798, Singapore; 6 Pervasive Technology Institute, Indiana University, Bloomington, IN 47405, USA; 7 Environmental Agency, Pekanbaru City, Riau Province, Indonesia; 8 School of Earth and Environment, University of Leeds, Leeds, LS2 9JT, UK

## Abstract

Vegetation and peatland fires cause poor air quality and thousands of premature deaths across densely populated regions in Equatorial Asia. Strong El-Niño and positive Indian Ocean Dipole conditions are associated with an increase in the frequency and intensity of wildfires in Indonesia and Borneo, enhancing population exposure to hazardous concentrations of smoke and air pollutants. Here we investigate the impact on air quality and population exposure of wildfires in Equatorial Asia during Fall 2015, which were the largest over the past two decades. We performed high-resolution simulations using the Weather Research and Forecasting model with Chemistry based on a new fire emission product. The model captures the spatio-temporal variability of extreme pollution episodes relative to space- and ground-based observations and allows for identification of pollution sources and transport over Equatorial Asia. We calculate that high particulate matter concentrations from fires during Fall 2015 were responsible for persistent exposure of 69 million people to unhealthy air quality conditions. Short-term exposure to this pollution may have caused 11,880 (6,153–17,270) excess mortalities. Results from this research provide decision-relevant information to policy makers regarding the impact of land use changes and human driven deforestation on fire frequency and population exposure to degraded air quality.

Vegetation and peatland fires are a common occurrence across Equatorial Asia^1^,^2^. Fires are used to manage the land, clear vegetation and to prepare and maintain land for agriculture^3^,^4^,^5^,^6^. Fires emit trace gases and fine particulate matter to the atmosphere causing extremely poor regional air quality^7^,^8^. Exposure of the population to degraded air quality results in thousands of premature deaths each year across Equatorial Asia^9^,^10^.

Whilst fires across Equatorial Asia have largely anthropogenic ignitions, with most burning occurring on deforested land^11^ and degraded peatlands^12^, the susceptibility of the landscape to fire is exacerbated by drought conditions during positive phases of the El Niño Southern Oscillation (ENSO)^13^,^14^,^15^ and the Indian Ocean Dipole (IOD)^16^. Across Borneo, fire emissions in El Niño years can be up to 30 times greater than during La Niña^17^.

In September and October 2015, strong positive ENSO and IOD conditions suppressed precipitation over Indonesia resulting in a dry and highly flammable landscape and widespread fires^18^. These fires caused the largest emissions of carbon dioxide from Equatorial Asia since the El Niño fires of 1997^4^,^19^,^20^ and resulted in a large regional haze event. Total particulate matter emissions from this region were 1.8 Tg over the September-October period, 2.2 times the 2002–2014 mean (Figure S1a), suggesting that the exposure to particulate pollution was substantially greater than in other years. In Singapore (5.5 million people, Department of Statistics, Singapore) the Pollutant Standards Index (PSI)^21^, used to indicate the impact of ambient air pollution on human health, reported unhealthy conditions (PSI: 101–200) for more than 50% of days in September-October 2015, with shorter periods of very unhealthy (PSI: 201–300) and hazardous (PSI > 300) conditions. However, the extent of the regional haze and the number of exposed people across the densely populated Equatorial Asia (Fig. 1a) is not accurately quantified due to the lack of high-resolution data in space and time either from remote sensing platforms or from numerical model simulations over the region.

In this work we quantify population exposure to degraded air quality conditions and associated mortality, which are mostly dictated by high concentrations of particulate matter with aerodynamic diameter less than 2.5 μm ([PM_2.5_]) attributable to the September-October 2015 wildfires. Our analysis is based on regional simulations of the Weather Research and Forecasting model with Chemistry (WRF-Chem)^22^,^23^ (Table S1) and an updated version of the Fire Inventory for NCAR^24^ (FINN v2, Fig. 1b and Figure S1b). The model is resolved at 10 km horizontal grid spacing and provides hourly output of key meteorological and chemical variables, thus overcoming major limitations associated with the use of coarse resolution global models for analogous assessments (~200 km and daily output)^8^,^10^,^25^. We show that high-resolution simulations can accurately describe the spatial and temporal distribution of harmful air pollutants in the region as a result of fires and allow for a detailed estimate of the total population exposure to unhealthy air quality conditions and of the associated mortality.

## Results and Discussion

We evaluate the model skill in reproducing spatio-temporal variability of aerosol optical properties and concentrations of particulate matter (PM) against a suite of space- and ground-based observations. WRF-Chem is able to simulate the spatial distribution of observed aerosol optical depth (AOD), with spatial correlation coefficients between weekly average AOD fields from MODIS (Terra and Aqua) and WRF-Chem ranging from 0.56–0.73 (Figure S2). The model exhibits highest correlations with observations during September-October when fires were active and extreme pollution episodes occurred (Figure S1b), with lower correlations (~0.2) associated with the low AOD values recorded in November. The model is characterized by a systematic negative bias in comparison to MODIS, with a Normalized Mean Bias Factor (NMBF, see Methods) of −0.78 and −0.54 for weekly averaged AOD collected onboard Terra and Aqua, respectively. Model underestimation of observed AOD in regions impacted by fires has been reported by numerous previous studies^7^,^8^,^10^,^26^. Many of these previous studies increased emissions to match observed AOD, although uncertainties in aerosol optical properties and water uptake could contribute to model-observation discrepancy of AOD^27^. Despite the underestimation in AOD magnitude, the spatial pattern of simulated AOD values does not present any systematic bias after long-range transport as indicated by the absence of sharp gradients in the ratio of simulated and observed AOD away from fire sources (Figure S2(d) and (h)). This thus increases the confidence in our simulated spatial patterns of AOD and hence our estimated regions of unhealthy air quality conditions.

**Figure 1:**
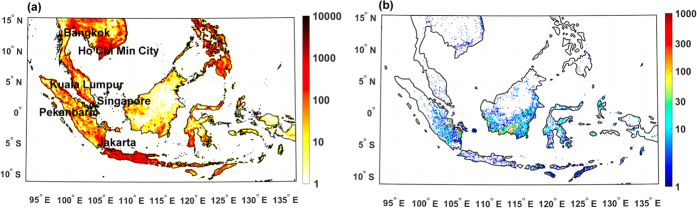
Population and fire location in Equatorial Asia. (**a**) 2013 population density (people km^−2^) in the model simulation area from LandScan High Resolution global Population Data at ~1 km × 1 km resolution^29^. The major cities mentioned in the text are also reported. (**b**) Mean total daily emissions of PM_2.5_ [μg m^−2^ s^−1^] from fires (FINN v2) during September-October. Maps created using Matlab vR2014b mapping toolbox http://www.mathworks.com/products/matlab/.

**Figure 2:**
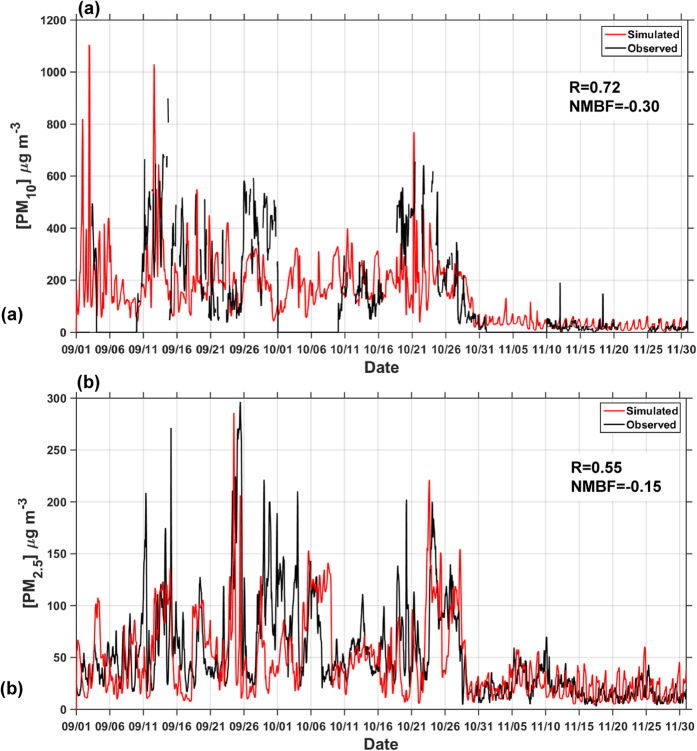
Observed and simulated particulate matter concentrations. Observed (black) and simulated (red) (**a**) 30-minute [PM_10_] at the ground-based station of Pekanbaru (Fig. 1a), in Sumatra and (**b**) hourly [PM_2.5_] averaged over Singapore. Summary statistics of model skill in reproducing daily mean concentrations are also reported in terms of correlation coefficient (R) and Normalized Mean Bias Factor (NMBF)^49^.

To further examine the ability of the model to reproduce regional air quality we compared WRF-Chem output against two ground-based sites measuring concentrations of particulate matter. The model captures well both the magnitude and temporal variability of surface particulate matter concentrations measured over Singapore and Pekanbaru in Sumatra (Tables S2 and S3, Figs 1a and 2). Analyses of high frequency (30 minute to 1 hr) measurements collected at those sites reveal peak PM_10_ (i.e. particulate matter with aerodynamic diameter less than 10 μm) concentrations above 600 μg m^−3^ in Pekanbaru and peak PM_2.5_ concentrations above 200 μg m^−3^ in Singapore, correctly simulated both in terms of magnitude and temporal occurrence (Fig. 2). The mean observed [PM_2.5_] in Singapore during September-November 2015 was 52 μg m^−3^, well reproduced by the model (45 μg m^−3^, NMBF = −0.15; Table S3). During this period, the temporal variability of PM_2.5_ shows a correlation coefficient (R) of 0.45 between hourly observations and simulated values (R = 0.55 for daily mean concentrations, Table S3 and Fig. 2b). Similar skills are found in Pekanbaru, where the mean observed [PM_10_] was 174 μg m^−3^, slightly underestimated by the model (140 μg m^−3^, NMBF = −0.24) and R = 0.57 (R = 0.72 for daily aggregated data). The higher model skill (lower underestimation) in describing PM_2.5_ concentrations than AOD is likely due to the issues in representation of water uptake and other aspects of the AOD calculation in the adopted aerosol scheme. The close agreement between observed and simulated PM concentrations suggests that for simulation of surface particulate air quality, FINN fire emissions do not need to be scaled during this period. At the end of October the onset of seasonal rains extinguished fires across the region^19^ (Figure S1b), hence particulate matter concentrations observed in November when few fires occurred were much lower (19 μg m^−3^ in Singapore), with this transition reproduced by the model (21 μg m^−3^).

**Figure 3:**
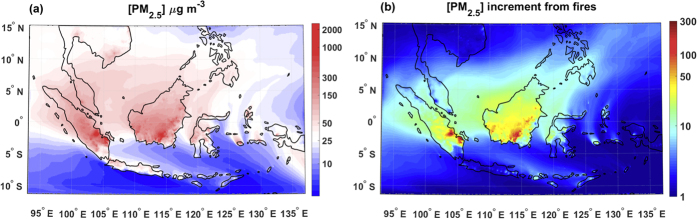
Contribution of fires to PM_2.5_. (**a**) Mean [PM_2.5_] in μg m^−3^ during September-October. The white shading indicates areas with concentrations corresponding to the WHO air quality guidelines for 24-hr [PM_2.5_] (i.e. 25 μg m^−3^)^28^, the blue shading refers to values below the limit and the red shading to concentrations above that limit. (**b**) 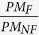, factor increase of [PM_2.5_] due to fires relative to background concentrations from other sources. PM_F_ and PM_NF_ are mean [PM_2.5_] concentrations during September-October of the run with fires and the one without fire emissions, respectively. Maps created using Matlab vR2014b mapping toolbox http://www.mathworks.com/products/matlab/.

All regions in Singapore observed mean [PM_2.5_] above the World Health Organization (WHO) air quality guidelines for 24-hr [PM_2.5_] (25 μg m^−3^)^28^, with highest concentrations in the Western and Southern regions (Table S3). Similarly in Pekanbaru, observed [PM_10_] was considerably above the WHO air quality guidelines for 24-hr [PM_10_] of 50 μg m^−3^. Simulated mean surface PM_2.5_ and PM_10_ concentrations exceeded the WHO air quality during September to October 2015, almost everywhere across Equatorial Asia (Fig. 3a and Figure S3a). To estimate the impact of this regional pollution, we calculate the number of people exposed to PM concentrations above the WHO 24-hr guidelines for at least 50% of September to October 2015. We find that 185 million people were persistently exposed to [PM_10_] higher than the WHO 24-hr guidelines and 217 million people exposed to [PM_2.5_] higher than the WHO 24-hr guidelines (Figure S4). We quantify the contribution of wildfires in degrading air quality by comparing simulations including fire emissions and an analogous run without fires. Wildfires fires during September and October are responsible for increasing background PM concentrations over most areas by at least a factor of 30, and up to a factor of 100 over regions over the eastern part of the provinces of Jambi and South Sumatra, and Central Kalimantan (Fig. 3b and Figure S3b). In the absence of fires, 73 million people in the urban areas of Jakarta, Ho Chi Minh City, Bangkok, Kuala Lumpur and Singapore, would have received persistent exposure to both [PM_10_] and [PM_2.5_] above WHO guidelines (Figure S4).

**Figure 4:**
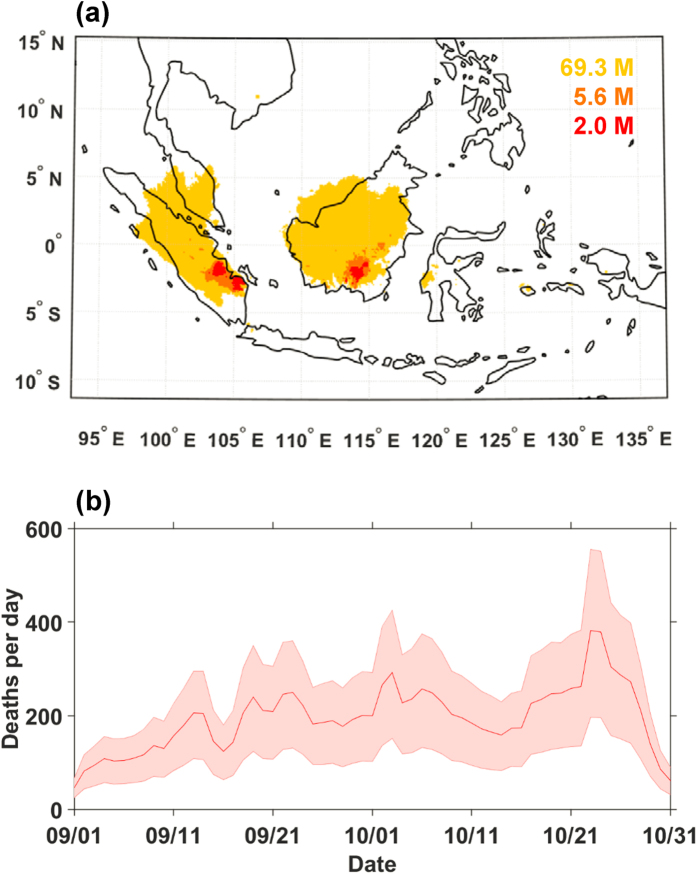
Human exposure to unhealthy air quality and premature deaths. (**a**) Areas of unhealthy (yellow, PSI > 100), very unhealthy (orange, PSI > 200) and hazardous (red, PSI > 300) air quality conditions on at least one day in two during September-October. The colored numbers refer to the total number of people (M = million) exposed to those different thresholds. For comparison, when only anthropogenic emissions are present, the number of people exposed to unhealthy conditions is approximately 4 million and no people are exposed to very unhealthy or hazardous conditions. Map created using Matlab vR2014b mapping toolbox http://www.mathworks.com/products/matlab/. (**b**) Estimated increase in premature deaths due to short-term exposure to [PM_2.5_] from wildfires during September-October (the pink shading indicates the 95% confidence interval). The total number of fatalities estimated is 11,880 (6,153–17,270) people (see Methods for details).

To further quantify the human population exposure to degraded air quality conditions, we calculate PSI values from simulated pollutant concentrations (see Methods) and estimate the number of people living in regions where the PSI is classified as unhealthy for at least 50% of the September-October 2015 period (Fig. 4a)^29^. Unhealthy PSI on at least one day in two occurred over most of Sumatra, Borneo, Malaysia and Singapore (consistently with Singapore PSI reports^21^) and very unhealthy and hazardous PSI were experienced in regions over Jambi and Palembang in Sumatra and Central Kalimantan (Fig. 4a). We calculate that in total 69 million people were exposed to unhealthy PSI levels, and that 6 million and 2 million people were exposed to very unhealthy and hazardous conditions for 50% of the period, respectively (Fig. 4a). Simulations without fire emissions indicate that only 4 million people, concentrated within the urban areas of Jakarta, Kuala Lumpur, and Ho Chi Minh City, would have been exposed to unhealthy PSI levels. Thus, our work indicates fires are responsible for increasing population exposure by more than factor 15, resulting in an additional 65 million people exposed to unhealthy conditions. The discrepancy in population exposure estimated using PSI relative to the WHO 24-hr limits (Figs 4 and S4) is attributable to the less restrictive bounds for unhealthy conditions in the PSI definition (Table S4).

We calculate excess all-cause mortality due to short-term exposure to PM_2.5_ using simulated 24 hr [PM_2.5_] and exposure-response functions from the most recent and comprehensive epidemiological studies linking health impacts to short-term exposure to outdoor fine particulate matter^30^,^31^ (see Methods). We apply a short-term exposure-response function because the population was exposed to high levels of pollution for <60 days and emissions during this period were substantially greater than usual (Figure S1). We estimate an additional 11,880 (with a 95% confidence interval of 6,153–17,270) all-cause premature mortalities due to short-term exposure to high concentrations of PM_2.5_ associated with wildfires during September-October 2015 (Figs 4b and S5a). This number of premature deaths is a conservative estimate since it is inferred from particulate matter concentrations that are slightly underestimated in our model simulations. Further, the estimated deaths represent only a fraction of the overall premature fatalities due to long-term exposure to unhealthy air quality conditions.

We have shown that a high-resolution regional atmospheric model in combination with satellite-derived fire emissions can provide a reliable assessment of air quality conditions during an intense air pollution episode caused by landscape fires. Our work confirms that the Fall 2015 Indonesian fires resulted in regional scale air pollution, with 69 million people exposed to persistent poor air quality, equivalent to 24% of the combined population of Malaysia, Singapore and Indonesia. Further, we estimate that 11,880 fatalities occurred as a result of short-term exposure to extreme particulate matter concentrations. If fires similar to those of Fall 2015 were to become more frequent, either due to changes in climate or through expansion of oil palm and timber concessions^12^, the public health burden from air pollution would rise considerably. We estimate that ~75,600 excess premature mortalities (`Figure S5b and Table S5, see Methods) would occur each year if the population received long-term exposure to the pollutant concentrations experienced in Fall 2015, consistent with a previous estimate^32^. Fire mitigation and control measures need to be implemented to prevent such episodes occurring in the future^18^.

## Methods

### WRF-Chem simulations

We applied the Weather Research and Forecasting model (version 3.5) with Chemistry (WRF-Chem)^23^ at 10 km horizontal resolution, with 51 vertical levels, over Equatorial Asia from 1 September to 1 December 2015. The domain is centered at (115°E, 2°N) and extends over 490 × 300 grid cells (longitudes × latitudes). A detailed summary of the physical and chemical schemes applied is provided in Table S1. Meteorological and chemical lateral boundary conditions are specified every 6 hours using output from the high resolution European Centre for Medium-Range Weather Forecasts (HRES-ECMWF) model at ~16 km^33^, and MOZART-4 (Model for Ozone and Related chemical Tracers, version 4)^34^. Climatological dust fields within MOZART-4 are replaced with CAM-Chem (Community Atmosphere Model with Chemistry) dust which are computed according to model simulated wind speeds and surface conditions^35^. To constrain the meteorology we use Four-Dimensional Data Assimilation (FDDA)^36^ to analysis-nudge model water vapor, wind and temperature fields above the boundary layer, with updates from ECMWF data every 6 hours. Biogenic emissions are computed online with MEGAN (Model of Emissions of Gases and Aerosols from Nature) version 2.04^37^, whereas fire emissions are specified using the FINN (Fire INventory from NCAR)^24^ inventory version 2^38^. FINN provides daily global estimates of trace gases and particles emitted by open biomass burning at ~1 km resolution. The new FINNv2 computes fire area burned from the available fire detections in a novel way relative to FINNv1.5 and also includes updated emission factors^39^,^40^,^41^,^42^, fuel loadings, and year-specific land cover datasets. Non‐methane organic compound emissions were allocated to the lumped chemical species of the MOZART mechanism based on the updated emission factors. Given this work focuses on the health impacts which are inferred from aerosol surface concentrations, the adopted plume-rise parameterization is expected to have little impact on the estimated health burden, as it largely affects the vertical distribution of aerosols, particularly in the upper troposphere^43^. Anthropogenic emissions are updated on a monthly basis using the EDGAR-HTAP V2.0^44^ inventory for 2010, which incorporates EDGAR 4.3 global emissions with the Regional Emission inventory in ASia (REAS)^45^ version 2.1, where available, on a 0.1° ×  0.1° grid resolution. The MOZART gas-phase chemistry is coupled with the GOCART (Global Ozone Chemistry Aerosol Radiation and Transport)^46^ bulk aerosol approach to reduce the computational cost. The aerosol direct and indirect feedbacks are turned off.

### Observations

Daily observations of Aerosol Optical Depth (AOD) at a wavelength of 550 nm collected by the MODerate resolution Imaging Spectroradiometer (MODIS) instruments onboard the Terra and Aqua satellites are used to evaluate model skills in reproducing the spatio-temporal patterns of intense pollution episodes associated with fires. Level-2 MODIS Collection-6 data, which have a resolution of 10 × 10 km (at nadir) for both Land and Ocean, are used in this study^47^. In the evaluation, daily values from WRF-Chem are extracted at the overpass hour (~10:30 and ~13:30 local solar time for MODIS onboard Terra and Aqua, respectively) and only pixels with simultaneous cloud free conditions in both MODIS and the model are considered when making the comparison with Taylor diagrams (see Figure S2 and section below on model evaluation for more details).

Simulated particulate matter concentrations are evaluated relative to ground-based measurements over Singapore and Sumatra. Hourly [PM_2.5_] from Singapore are collected by the National Environment Agency (NEA) using a Thermo Scientific™ 5030 SHARP Monitor over five regions (Table S2) during September-November 2015 and have been accessed from the National Environment Agency website^21^. Model evaluation is conducted by averaging the grid cells that included the five regions defined by NEA. NEA also provides an hourly Pollutant Standards Index (PSI, defined in the next section)^21^, which was used in this study to evaluate threshold exceedances simulated by the model.

30-min [PM_10_] measured at Pekanbaru (101.45 E, 0.51 N) in the Riau region using a Met One BAM 1020, Real-Time Portable Beta Attenuation Mass Monitor (BAM-1020) were analyzed for the entire simulation period.

For both Singapore and Sumatra, model skill was quantified based on PM hourly and daily means, selecting only hours with simultaneous data available between observations and model simulations.

### Population data and Pollutant Standards Index (PSI)

Population exposure to degraded air quality conditions and premature deaths are estimated based on the 2013 LandScan High Resolution global Population Data product that provides population density data gridded with a resolution of 30 arc-seconds (approximately 1 km at the equator)^29^. The population density data have been upscaled to match the WRF-Chem grid, averaging over cells of size 0.1 degrees over latitude and longitude. The final population data are then obtained by multiplying the population density by each grid cell surface area. The total population over the analyzed domain is ~488 million people.

Population exposure is quantified based on exceedances of the Pollutant Standards Index (PSI), which allows assessment of air quality conditions based on six criteria pollutant concentrations and classifies air quality at different levels (from good to hazardous)^21^.

The PSI is defined as:


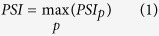


where 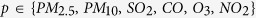, and the PSI for each pollutant *p* is obtained via linear interpolation of the observed concentration *C*_*p*_ from predefined blocks *C*_*p,b*_ (see Table S4 for their definition):


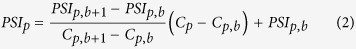


with 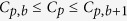.

### Statistical metrics of model performance

We use Taylor diagrams^48^ to compare spatial patterns of weekly averaged AOD fields from MODIS observations and WRF-Chem simulations. Taylor diagrams provide information on the spatial correlation coefficient (R), the Root Mean Squared Difference that is proportional to the distance of a point to a reference on the x-axis, and the ratio of spatial standard deviations between observations and simulated values.

We also assess model performance in reproducing hourly observations of particulate matter concentrations in terms of Normalized Mean Bias Fraction (NMBF), which is a symmetric and unbiased metric^49^:


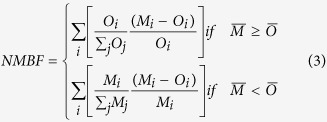


where O and M refer to observations and output from WRF-Chem simulations respectively, and 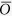 and 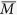 are the associated means. *i* and *j* vary between 1 and the total number of observations/output analyzed. A positive NMFB indicates that the model overestimates observations by a factor 1 + NMBF, whereas if it is negative WRF-Chem underestimates observations by a factor 1-NMBF.

### Mortality estimates

Estimation of all-cause mortality for the overall population due to short-term exposure to increased PM_2.5_ from fires is performed using simulated surface-level [PM_2.5_] in combination with an exposure-response function^31^. We calculate the Relative Risk (RR) due to short-term exposure as:


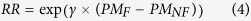


where *PM*_*F*_ and *PM*_*NF*_ are the daily PM_2.5_ concentrations (in μg m^−3^) in the run with fire and no fire emissions respectively, and *γ* is the excess mortality per unit increase in PM_2.5_. A recent meta-analysis of 110 peer-reviewed epidemiological short-term time-series studies of daily mortality and hospital admissions have estimated this parameter as 0.00104, with a 95% confidence interval of (0.00052, 0.00156)^30^. It is important to note that the RR meta-analysis by Atkinson *et al*.^30^ does not include epidemiological studies from Equatorial Asia since they don’t exist and it is mostly based on studies conducted in Europe and in the United States. This implies that short-term mortality estimates for Equatorial Asia (independently on the RR functional form adopted) are likely to be conservative as based on RR functions developed on lower pollutant levels than those observed in our region. The attributable fraction 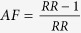 for every cell and every day is then computed, and the total mortality in each cell and each day is calculated as:


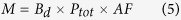


where *B*_*d*_ is the daily, country-specific baseline risk of deaths from noncommunicable diseases (NCD), obtained from the 2012 country-specific statistics from the WHO Global Health Observatory^50^ and *P*_*tot*_ is the total population for each grid point from the 2013 LandScan High Resolution global Population Data^29^.

To estimate premature mortality due to long-term exposure to wildfire PM_2.5_, we use the integrated exposure-response (IER) relationship^51^ that compiles epidemiological evidence across a wide range of PM concentrations from different combustion sources. The IER has been used in a number of recent studies^9^,^52^,^53^,^54^,^55^,^56^,^57^ and allows for age-dependent calculation of relative risk for five different diseases (RR_d_) associated with PM_2.5_ exposure: lower respiratory infections, chronic obstructive pulmonary disease, lung cancer, ischemic heart disease and stroke.

We estimated wildfire contribution to premature mortality due to long-term exposure to PM_2.5_ using the RR lookup table and AF function in^53^:


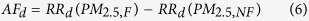


where PM_2.5,F_ and PM_2.5,NF_ refer to the annual mean PM_2.5_ concentrations in μg m^−3^ in the run including fires and the control run without fires, respectively. The annual mean for the run including fires was estimated assuming that only two months (i.e. September and October) contributed to fire emissions and that November is representative of PM concentrations during the months without fires. The total mortality can be then calculated by applying Equation 5. Cause-specific background disease rates for the Southeast Asian region are taken from Global Burden of Disease 2013 assessment^58^ for the year 2013 (latest year available), while population age-group structures are taken from^55^ for the year 2010 (latest year available).

## Additional Information

**How to cite this article**: Crippa, P. *et al*. Population exposure to hazardous air quality due to the 2015 fires in Equatorial Asia. *Sci. Rep.* **6**, 37074; doi: 10.1038/srep37074 (2016).

**Publisher's note**: Springer Nature remains neutral with regard to jurisdictional claims in published maps and institutional affiliations.

## Supplementary Material

Supplementary Information
