# Supplementary material for: Population exposure to hazardous air quality due to the 2015 fires in Equatorial Asia
Source: Sci Rep. 2016 Nov 16;6:37074. doi: 10.1038/srep37074 (PMC5111049; doi:10.1038/srep37074)
Supplement: Supplementary Information [file srep37074-s1.pdf]

Supplementary Information to:

Population exposure to hazardous air quality due to the 2015 fires in Equatorial Asia

P. Crippa[1*], S. Castruccio[2], S. Archer-Nicholls[3], G. B. Lebron[4], M. Kuwata[4,5], A. Thota[6], S. Sumin[7], E. Butt[8], C. Wiedinmyer[3], D. V. Spracklen[8]

[1]COMET, School of Civil Engineering and Geosciences, Newcastle University, Newcastle upon Tyne, NE1 7RU, UK

[2]School of Mathematics and Statistics, Newcastle University, Newcastle upon Tyne, NE1 7RU, UK

[3]Atmospheric Chemistry Observations & Modeling Laboratory, National Center for Atmospheric Research, Boulder, CO, 80301, USA

[4]Earth Observatory of Singapore, Nanyang Technological University, Singapore, 639798.

[5]Asian School of the Environment, Nanyang Technological University, Singapore, 639798.

[6]Pervasive Technology Institute, Indiana University, Bloomington, IN 47405, USA

[7]Environmental Agency, Pekanbaru City, Riau Province, Indonesia

[8]School of Earth and Environment, University of Leeds, Leeds, LS2 9JT, UK

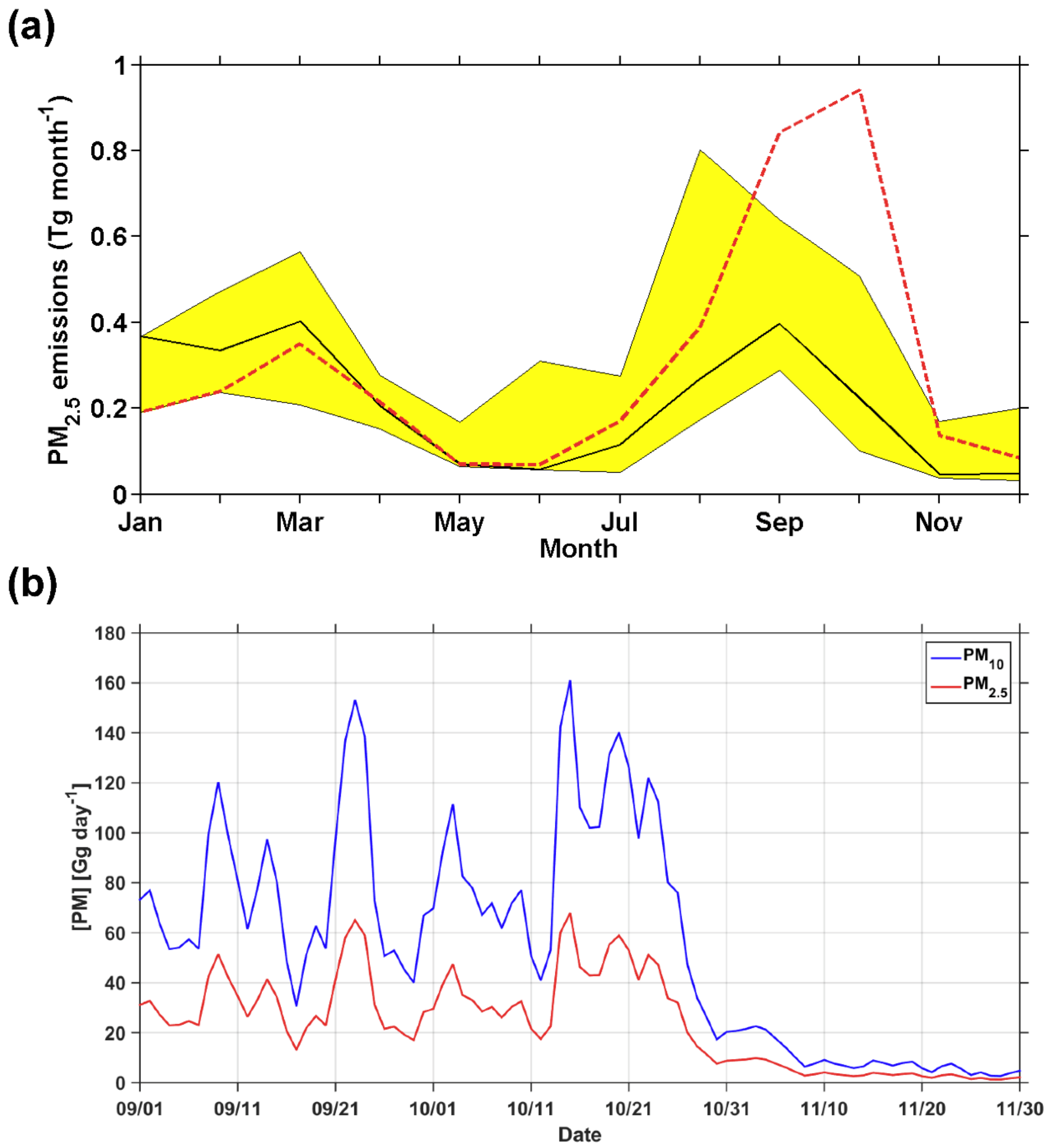

Figure S1: Particulate matter emissions from fires. (a) Functional boxplot of $PM_{2.5}$ monthly emissions (in Tg) from 2002 to 2015 over Equatorial Asia. The black line indicates the median emission profile during 2002-2014 and the yellow shading the interquartile range (25th to 75th functional percentile) over the same period, as defined in [1]. The dashed red line refers to the emissions in 2015. (b) Total daily mean particulate matter emissions from fires [Gg day^{-1}] (FINN v2) over the simulated domain during September-November 2015. At the end of October the onset of seasonal rains extinguished fires across the region.

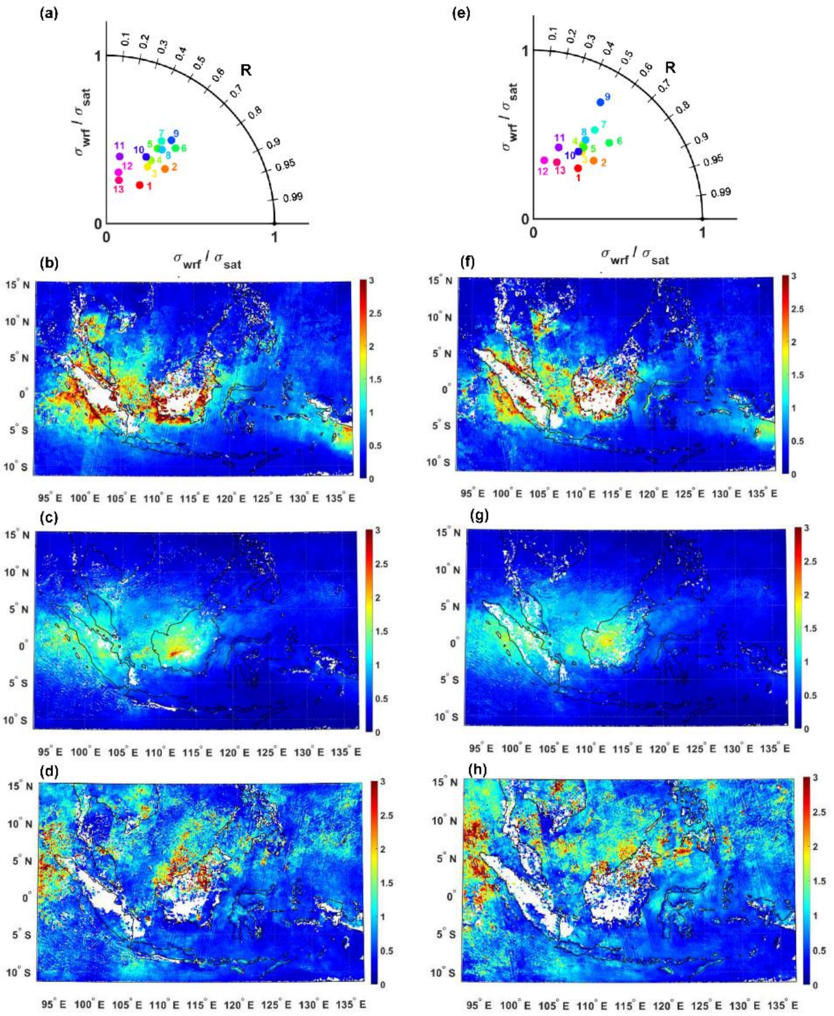

Figure S2: Simulated and observed aerosol optical depth. (a) and (e)Taylor diagram comparing the spatial fields of weekly mean aerosol optical depth (AOD) at a wavelength of 550nm from WRF-Chem and MODIS (Terra (first column) and Aqua (second column)). The numbers denote the week from the beginning of September 2015 (i.e., 1 refers to the mean AOD during 1-7 September). The weekly mean is computed for the overpass time and only grid cells with simultaneous cloud free conditions from observations and simulations are considered in the comparison. Panels (b-c) show the monthly mean AOD during October measured by MODIS onboard Terra and simulated by WRF-Chem respectively (panels (f-g) refer to the same comparison relative to MODIS onboard Aqua). The spatial mean Normalized Mean Bias Factor (i.e. NMBF, see Methods) of weekly averaged AOD are -0.78 and -0.54 for MODIS onboard Terra and Aqua, respectively. (d) and (h) ratio of simulated and observed AOD (i.e. panel (d) shows AOD in (c) divided by AOD (b) and panel (h) shows AOD in (g) divided by AOD (f). Maps created using Matlab vR2014b mapping toolbox http://www.mathworks.com/products/matlab/.

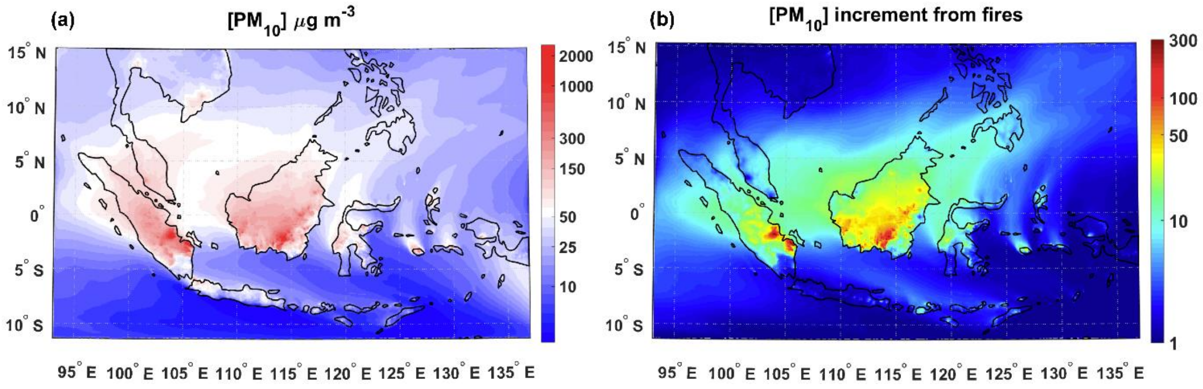

Figure S3: Contribution of fires to PM_{10}. (a) Mean [PM_{10}] in μg m^{-3} during September-October. The white shading indicates areas with concentrations corresponding to the WHO air quality guidelines for 24-hr [PM_{10}] (i.e. 50 μg m^{-3}) [2], the blue shading refers to values below the limit and the red shading to concentrations above that limit. (b) $\frac{PM_F}{PM_{NF}}$, factor increase of [PM_{10}] due to fires relative to background concentrations from other sources. PM_F and PM_{NF} are mean [PM_{10}] concentrations during September-October of the run with fires and the one without fire emissions, respectively. Maps created using Matlab vR2014b mapping toolbox http://www.mathworks.com/products/matlab/.

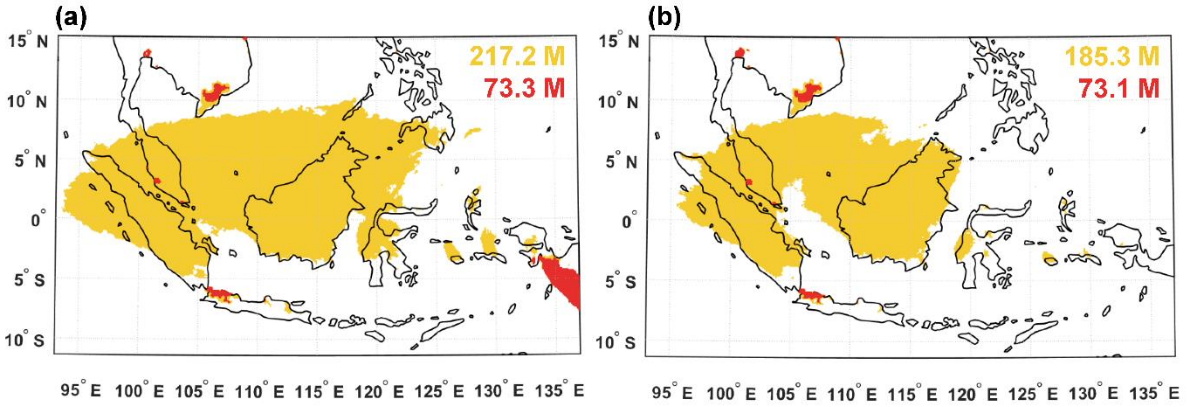

Figure S4: Human exposure to particulate matter above the WHO limits. Areas exceeding the 24-hr WHO limit for (a) [$PM_{2.5}$] and (b) [PM_{10}] on at least one day in two during September and October when only anthropogenic emissions are present (red shading) and when also fires are included (yellow shading). The numbers refer to the the total number of people exposed (M=million). Maps created using Matlab vR2014b mapping toolbox http://www.mathworks.com/products/matlab/.

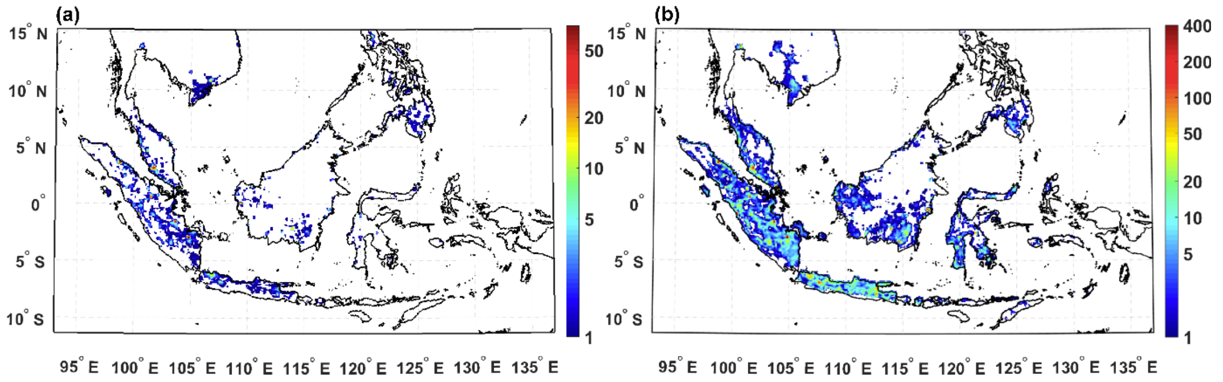

Figure S5: Premature deaths due to exposure to particulate matter from fires. Total excess premature deaths due to (a) short-term and (b) long-term exposure to high concentrations of $PM_{2.5}$ during September and October. See Methods for details on the computation. Map created using Matlab vR2014b mapping toolbox http://www.mathworks.com/products/matlab/.

Table S1: Physical and chemical schemes adopted in WRF-Chem simulations.

**Simulation settings**	**Values**
Simulation Period	01/09/2015-01/12/2015
Domain size	490 × 300 cells
Horizontal resolution	10 km
Vertical resolution	51 eta levels up to 50 hPa
BDY for meteorology	HRES-ECMWF at 16km × 16km
BDY for chemistry	MOZART driven by GEOS meteorology and CAM dust
**Physics option**	**Adopted scheme**
Microphysics	Morrison
Longwave/Shortwave Radiation	Rapid Radiative Transfer Model (RRTMG)
Surface layer	Monin Obhukov similarity
Land Surface	Noah Land Surface Model
Planetary boundary layer	Yonsei University scheme
Cumulus parameterizations	Grell-Freitas
**Chemistry option**	**Adopted scheme**
Photolysis	Madronich TUV
Gas-phase chemistry	MOZART
Aerosols	GOCART
Anthropogenic emissions	EDGAR HTAP v2.2
Biogenic emissions	MEGAN
Fire emissions	FINNv2

Table S2: Regions for Air Quality Reporting in Singapore [3].

Region	Town Centres /Areas
North	Admirality, Kranji, Woodlands, Sembawang, Yishun, Yio Chu Kang, Seletar, Sengkang
South	Holland, Queenstown, Bukit Merah, Telok Blangah, Pasir Panjang, Sentosa, Bukit Timah, Newton, Orchard, City, Marina South
East	Serangoon, Punggol, Hougang, Tampines, Pasir Ris, Loyang, Simei, Kallang, Katong, East Coast, Macpherson, Bedok, Pulau Ubin, Pulau Tekong
West	Lim Chu Kang, Choa Chu Kang, Bukit Panjang, Tuas, Jurong East, Jurong West, Jurong Industrial Estate, Bukit Batok, Hillview, West Coast, Clementi
Central	Thomson, Marymount, Sin Ming, Ang Mo Kio, Bishan, Serangoon Gardens, MacRitchie, Toa Payoh

Table S3: Model evaluation statistics. Summary statistics of model skill in reproducing [$PM_{2.5}$] over five regions in Singapore and the whole Singapore area, and [PM_{10}] in Pekanbaru. For each site some summary statistics and metrics of model performance based on hourly (hh) and daily (dd) averaged data are reported on different columns: mean (μ), standard deviation (σ), correlation coefficient (R), Normalized Mean Bias Factor (NMBF) and Normalized Mean Absolute Error Factor (NMAEF) [4]. The NMAEF is defined as: $NMAEF = \sum_i \frac{O_i}{\sum_j O_j} \frac{|M_i - O_i|}{O_i} \, if \, \overline{M} \geq \overline{O}$ and $NMAEF = \sum_i \frac{M_i}{\sum_j M_j} \frac{|M_i - O_i|}{M_i} \, if \, \overline{M} \leq \overline{O}$, where O and M refer to observations and output from WRF-Chem simulations respectively, and $\overline{O}$ and $\overline{M}$ are the associated means. *i* and *j* vary between 1 and the total number of observations/output analyzed.

	μ_{obs}	μ_{mod}	σ_{obs}	σ_{mod}	R	NMBF	NMAEF
North (hh)	50.60	42.05	44.32	38.23	0.44	-0.20	0.68
South (hh)	55.02	39.22	51.01	38.39	0.48	-0.40	0.79
East (hh)	51.22	39.65	45.41	36.32	0.46	-0.29	0.72
West (hh)	56.63	64.26	51.52	45.59	0.30	0.14	0.72
Central (hh)	45.05	42.05	38.91	38.23	0.44	-0.07	0.63
Singapore (hh)	51.70	45.12	46.64	37.04	0.45	-0.15	0.63
Pekanbaru (hh)	173.55	140.24	177.75	125.48	0.57	-0.24	0.70
North (dd)	50.60	42.05	37.59	32.90	0.57	-0.20	0.53
South (dd)	55.02	39.22	43.34	33.38	0.59	-0.40	0.65
East (dd)	51.22	39.65	38.26	31.64	0.57	-0.29	0.57
West (dd)	56.63	64.26	42.42	32.49	0.44	0.13	0.54
Central (dd)	45.05	42.05	32.46	32.90	0.56	-0.07	0.48
Singapore (dd)	51.70	45.12	38.57	31.95	0.55	-0.15	0.49
Pekanbaru (dd)	202.51	155.28	174.72	113.75	0.72	-0.30	0.58

Table S4: Pollutant Standards Index (PSI). PSI index break points as defined from the Singapore National Environment Agency [3] (*When 8-hour ozone concentration exceeds 785µg m^{-3}, the PSI sub-index is calculated using the 1-hour concentration; **Sub-index for nitrogen dioxide is reported only when the 1-hour concentration equals or exceeds 1130 µg m^{-3}).

Index Category	PSI	24-hr $PM_{2.5}$ (µg m^{-3})	24-hr PM_{10} (µg m^{-3})	24-hr SO_2 (µg m^{-3})	8-hr CO (mg m^{-3})	8-hr O_3 (µg m^{-3})	1-hr NO_2 (µg m^{-3}) **
Good	0 - 50	0 - 12	0 - 50	0 - 80	0 - 5.0	0 - 118	-
Moderate	51 - 100	13 - 55	51 - 150	81 - 365	5.1 - 10	119 - 157	-
Unhealthy	101 - 200	56 - 150	151 - 350	366 - 800	10.1 - 17.0	158 - 235	1130
Very Unhealthy	210 - 300	151 - 250	351 - 420	801 - 1600	17.1 - 34.0	236 – 785*	1131 - 2260
Hazardous	301 - 500	251 - 500	421 - 600	1601 - 2620	34.1 - 57.5	786 – 1180*	2261 - 3750

Table S5. Long-term excess premature mortality estimates. Cause-specific excess mortality due to long-term exposure to $PM_{2.5}$ concentrations in the year 2015. The number of deaths for COPD, LNC, IHD and stroke refer to the adult population (>25 years old), whereas LRI is computed for under-5 children, as in [5] (see Methods).

Disease	Excess mortality (# deaths)
Chronic Obstructive Pulmonary Disease (COPD)	3,384
Lung Cancer (LNG)	3,223
Ischemic Heart Disease (IHD)	2,492
Stroke	28,777
Low Respiratory Infections (LRI)	37,713
Total deaths	75,589

References

1 Sun, Y. & Genton, M. G. Functional Boxplots. *Journal of Computational and Graphical Statistics* **20**, 316-334, doi:10.1198/jcgs.2011.09224 (2011).

2 WHO. Air quality guidelines for particulate matter, ozone, nitrogen dioxide and sulfur dioxide - Global update 2005 - Summary of risk assessment. 22 (World Health Organization, 2006).

3 NEA. *National Environment Agency - Air Pollution Control*, <http://www.nea.gov.sg/anti-pollution-radiation-protection/air-pollution-control/psi> (2016).

4 Yu, S., Eder, B., Dennis, R., Chu, S.-H. & Schwartz, S. E. New unbiased symmetric metrics for evaluation of air quality models. *Atmospheric Science Letters* **7**, 26-34, doi:10.1002/asl.125 (2006).

5 Apte, J. S., Marshall, J. D., Cohen, A. J. & Brauer, M. Addressing Global Mortality from Ambient PM2.5. *Environmental Science & Technology* **49**, 8057-8066, doi:10.1021/acs.est.5b01236 (2015).
